# Retinoic Acid is Required for Normal Morphogenetic Movements During Gastrulation

**DOI:** 10.3389/fcell.2022.857230

**Published:** 2022-04-21

**Authors:** Michal Gur, Tamir Edri, Sally A. Moody, Abraham Fainsod

**Affiliations:** ^1^ Department of Developmental Biology and Cancer Research, Institute for Medical Research Israel-Canada, Faculty of Medicine, The Hebrew University of Jerusalem, Jerusalem, Israel; ^2^ Department of Anatomy and Cell Biology, School of Medicine and Health Sciences, George Washington University, Washington, DC, United States

**Keywords:** retinoic acid signaling, embryo development, gastrulation delay, *Xenopus* embryo, morphogenetic movements, Brachet’s cleft, tissue separation

## Abstract

Retinoic acid (RA) is a central regulatory signal that controls numerous developmental processes in vertebrate embryos. Although activation of *Hox* expression is considered one of the earliest functions of RA signaling in the embryo, there is evidence that embryos are poised to initiate RA signaling just before gastrulation begins, and manipulations of the RA pathway have been reported to show gastrulation defects. However, which aspects of gastrulation are affected have not been explored in detail. We previously showed that partial inhibition of RA biosynthesis causes a delay in the rostral migration of some of the earliest involuting cells, the leading edge mesendoderm (LEM) and the prechordal mesoderm (PCM). Here we identify several detrimental gastrulation defects resulting from inhibiting RA biosynthesis by three different treatments. RA reduction causes a delay in the progression through gastrulation as well as the rostral migration of the *goosecoid*-positive PCM cells. RA inhibition also hampered the elongation of explanted dorsal marginal zones, the compaction of the blastocoel, and the length of Brachet’s cleft, all of which indicate an effect on LEM/PCM migration. The cellular mechanisms underlying this deficit were shown to include a reduced deposition of fibronectin along Brachet’s cleft, the substrate for their migration, as well as impaired separation of the blastocoel roof and involuting mesoderm, which is important for the formation of Brachet’s cleft and successful LEM/PCM migration. We further show reduced non-canonical Wnt signaling activity and altered expression of genes in the Ephrin and PDGF signaling pathways, both of which are required for the rostral migration of the LEM/PCM, following RA reduction. Together, these experiments demonstrate that RA signaling performs a very early function critical for the progression of gastrulation morphogenetic movements.

## Introduction

Retinoic acid (RA) is a central regulatory signal controlling numerous developmental processes in vertebrate embryos, and it is a major contributor to tissue homeostasis in adults (Metzler and Sandell, 2016; le Maire and Bourguet, 2014; Nolte et al., 2019; Draut et al., 2019; Summerbell and Maden, 1990). RA is produced *in vivo* by two sequential oxidation reactions from vitamin A (retinol), first to retinaldehyde and subsequently to the acid form (Kedishvili, 2016; Shabtai and Fainsod, 2018; Blaner, 2019; Ghyselinck and Duester, 2019). Based on retinoid content analysis and mutant or RA-manipulated embryos, it was suggested that late blastula vertebrate embryos are poised to initiate RA signaling but it still requires the expression of a retinaldehyde dehydrogenase activity to finalize the biosynthesis of RA (Chen et al., 1994; Creech Kraft et al., 1994; Kraft et al., 1994; Niederreither et al., 1999; Begemann et al., 2001; Lloret-Vilaspasa et al., 2010; Zile, 2010). Premature RA signaling can be experimentally elicited by precocious expression of a retinaldehyde dehydrogenase (Ang and Duester, 1999). Retinoids including RA, have been detected in the gastrula organizer, called the Spemann-Mangold organizer in *Xenopus*, a central embryonic regulatory structure (Chen et al., 1992, 1994, 2001; Hogan et al., 1992; Creech Kraft et al., 1994; Kraft et al., 1994; Niederreither et al., 1997; Yelin et al., 2005). The early expression pattern of *aldehyde dehydrogenase 1a2* (*aldh1a2*; *raldh2*) is consistent with the suggestion that the retinaldehyde dehydrogenase encoded by this gene is central for RA biosynthesis in the embryo from gastrula stages (Niederreither et al., 1997; Begemann et al., 2001; Chen et al., 2001; Blentic et al., 2003; Halilagic et al., 2003; Liang et al., 2008; Shabtai et al., 2018), and in agreement, mutant *aldh1a2* embryos exhibit developmental defects soon after gastrulation and subsequently die (Niederreither et al., 1999; Begemann et al., 2001). Early expression of *aldh1a2* in the gastrula organizer has been identified in several vertebrate embryos (Chen et al., 2001; Blentic et al., 2003; Halilagic et al., 2003).

The onset of gastrulation involves a series of morphogenetic processes involving cell shape changes, cell rearrangements, and internalization of the mesoderm and endoderm that involves vegetal rotation, mesenchymal-like, and ameboid migration (Kaneda and Motoki, 2012; Huang and Winklbauer, 2018; Winklbauer, 2020). These extensive cell movements result in the reorganization of the embryo into the three primary germ layers (Fagotto, 2020; Keller and Sutherland, 2020). Close to the onset of gastrulation, the *Xenopus* embryo has a multilayered epithelium on the animal side that partially envelopes the blastocoel to form the blastocoel roof (BCR). On the vegetal side, the blastocoel is surrounded by the marginal zone (MZ) and the endoderm (Kaneda and Motoki, 2012; Huang and Winklbauer, 2018; Winklbauer, 2020). Involution of the MZ forms the mesodermal and endodermal germ layers during gastrulation and involves an inward folding of the MZ around the BCR to form the blastopore, starting from the dorsal side. This involution event creates an apposition of the mesendodermal layer and the BCR separated by a small gap, Brachet’s cleft (Keller et al., 2003; Gorny and Steinbeisser, 2012). Formation of Brachet’s cleft separating the internalized mesendoderm and the enveloping ectoderm involves multiple signals including the Wnt receptor Frizzled 7 (Winklbauer et al., 2001; Köster et al., 2010; Kraft et al., 2012; Brinkmann et al., 2016; Lee et al., 2016). The involuted mesendodermal cells migrate towards the animal pole using the BCR as the substrate (Keller et al., 2003). As the involution extends all around the blastopore, migration of the internalized mesendoderm towards the prospective rostral region results in compaction of the blastocoel (Keller et al., 2003).

Classically, activation of *Hox* expression is considered one of the earliest functions of RA signaling in the embryo (Lloret-Vilaspasa et al., 2010; Neijts and Deschamps, 2017; Nolte et al., 2019). However, a number of studies have reported very early functions of RA, close to the onset of gastrulation (Yelin et al., 2005; Janesick et al., 2018; Shukrun et al., 2019). We previously described that as a result of partial inhibition of RA biosynthesis, we observed a delay in the rostral migration of some of the earliest involuting cells, the leading edge mesendoderm (LEM) and the prechordal mesoderm (PCM) (Yelin et al., 2007). Here we identified several detrimental gastrulation defects resulting from inhibiting RA biosynthesis. Inhibition of RA signaling induces a delay in the progression through gastrulation and a delay in the rostral migration of the *goosecoid*-positive PCM cells after their involution. Supporting this conclusion, we observe that RA inhibition hampers the elongation of explanted dorsal MZs (DMZs). Analysis of several gastrulation processes important for rostral migration of the LEM/PCM cells revealed that reduced RA signaling reduces the deposition of fibronectin along Brachet’s cleft. The tissue separation behavior important for Brachet’s cleft formation is also reduced when RA biosynthesis is inhibited. Abnormal morphogenetic movements were observed in manipulated embryos affecting the position of the neural plate, the compaction of the blastocoel, and the length of Brachet’s cleft. These results show that in *Xenopus* embryos, RA signaling performs a very early function important for the early progression of gastrulation morphogenetic movements.

## Materials and Methods

### Embryo Culture and Treatments

*Xenopus laevis* frogs were purchased from Xenopus 1 or Nasco (Dexter, MI or Fort Atkinson, WI, United States). Experiments were performed after approval and under the supervision of the Institutional Animal Care and Use Committee (IACUC) of the Hebrew University (Ethics approval no. MD-17-15281-3) and the George Washington University (Ethics approval no. A233). Embryos were obtained by *in vitro* fertilization, incubated in 0.1% Modified Barth’s Solution and Hepes (MBSH), and staged according to (Nieuwkoop and Faber, 1967). Treatments with 4-Diethylaminobenzaldehyde (DEAB, Sigma, dissolved in DMSO) or 3,7-Dimethyl-2,6-octadienal (citral, Aldrich, diluted in EtOH), were performed in 0.1% MBSH from the midblastula transition (MBT, stage 8.5) until the desired stage for analysis.

Whenever necessary, embryos were injected at the one to four cell stage with *in vitro* transcribed capped mRNA or the ATF2 reporter plasmid. Capped mRNAs were prepared using the appropriate RNA polymerase. Cap analog (m7G (5′)ppp (5′)G; New England Biolabs, USA) was added to the reaction mixture using a cap:GTP ratio of 5:1. Expression plasmids were linearized and transcribed as previously described: *cyp26a1* (Hollemann et al., 1998); dominant-negative *frizzled7* (dnfzd7) (Winklbauer et al., 2001); *ß-galactosidase* (Yan et al., 2009).

### Quantitative Reverse Transcription Real-Time PCR (qPCR)

Total RNA from embryos was extracted with the Aurum Total RNA Mini Kit (Bio-Rad), and cDNA was synthesized using iScript cDNA Synthesis Kit (Bio-Rad). The real-time PCR reactions were performed using the CFX384 Real-Time System (Bio-Rad) and iTaq universal SYBR Green Supermix (Bio-Rad). Each experiment was repeated at least three independent times and each time the samples were run in triplicate. GAPDH was used as the housekeeping reference gene. The primers used for qPCR analysis are listed in Table 1.

**TABLE 1:** Primers for qPCR analysis.

Gene	Forward Primer	Reverse Primer
*efnb1.L*	TCA​CAT​GGA​ACT​CGC​AGA​A	AGT​ATT​CAT​AAG​GCT​GGG​AAG​AG
*efnb2.S*	GAT​CCG​AGG​TGG​CCT​TAT​TT	CAA​CAA​CAG​CAC​AAC​AAG​AGT​G
*efnb3.S*	CCT​CTA​CCA​ATC​TCC​CAT​GTT​C	GCA​GAC​CCA​TCC​CAA​TAC​TC
*epha4.L/S*	GTG​GTG​CTG​ATG​GAG​AGT​G	TCT​GTT​GAG​AGG​GCT​TTG​TAG
*ephb4.L/S*	CTG​GCT​CCT​CCT​CCT​GTG​T	CCC​ACT​GTC​CGT​CCA​CTT​T
*pdch8.L/S*	AAT​CTG​GTC​GCC​TCA​CTC​TT	ATG​ACT​CGC​ACG​ATG​ACT​TT
*pdgfa.L/S*	GTC​AAG​TGC​CAG​CCA​TCA​A	GAT​GTT​CCT​CTA​ACC​GCA​CAA
*pdgfra.L*	GCT​GCT​GTC​TTG​GTC​CTT​CT	TTA​CTC​GCC​ATC​TTA​TTT​CAT​ACC

### Whole-Mount *in situ* Hybridization

Whole-mount *in situ* hybridization and double *in situ* hybridization were performed as previously described (Epstein et al., 1997). Embryos treated with DEAB (300 µM) or citral (70 µM) as well as untreated siblings, were fixed at stage 15 in 4% paraformaldehyde in MEM buffer (0.1 M MOPS, 0.5 M NaCl, 1 mM EGTA, 2 mM MgSO_4_), and processed for whole-mount *in situ* hybridization. Probes were prepared by *in vitro* transcription using Digoxigenin or Fluorescein labeling mix (Roche). Probes were transcribed as previously described: *gsc* (*goosecoid*) (Cho et al., 1991), *sox3* (Penzel et al., 1997)*.*

### Fibronectin (FN) Immunodetection

Embryos treated with DEAB (300 µM), citral (70 µM) or diluent alone, or embryos microinjected at cleavage stages with *cyp26a1* mRNA, as well as untreated, control siblings, were fixed when controls reached stage 10.5 in 4% paraformaldehyde in MEM buffer, washed and bisected along the midsagittal plane. Embryos were processed for immunohistochemical detection of fibronectin according to Davidson et al. (2004) using a mouse anti-fibronectin monoclonal antibody (1 μg/ml; Developmental Hybridoma Bank #4H2) and goat anti-mouse HRP-conjugated IgG (1:250, Cell Signaling #7076). After the diaminobenzidine chromogen reaction, post-fixed bisected embryos were photographed using the cellSens program on an Olympus SZX16 stereomicroscope.

### Measurements of Gastrula Morphology

Embryos were treated with citral (70 µM) or diluent alone, as above. The treated embryos and their untreated, control siblings were fixed when controls reached stage 10.5 as above. Embryos were bisected along the midsagittal plane and the width of the floor of the blastocoele, the length of Brachet’s cleft, and the length of the archenteron were measured using the cellSens program on an Olympus SZX16 stereomicroscope.

### Lineage Tracing of Blastomere Clones

Embryos were chosen at the 2-cell stage if the first cleavage furrow bisected the grey crescent in order to accurately identify the dorsal-ventral axis (Klein, 1987; Moody, 2018). When selected embryos reached the 32-cell stage, a single blastomere of known fate (Dale and Slack, 1987; Moody, 1987) was microinjected with 100 pg of nuclear-localizing *ß-galactosidase* mRNA. Embryos were then treated with citral (70 µM) or diluent alone from blastula stages, and harvested when untreated, control siblings reached appropriate stages. They were fixed as above, processed for the histochemical detection of ß-Galactosidase activity, as previously described (Yan et al., 2009), and the position of the labeled descendant cells mapped as previously described (Dale and Slack, 1987; Moody, 1987; Bauer et al., 1994).

### Tissue Separation Behavior Assay

Embryos were treated with DEAB (300 µM), citral (70 µM) or diluent alone, or microinjected at cleavage stages with *cyp26a1* mRNA. When their untreated, control siblings reached stage 10+, the blastocoel roof (BCR) and the anterior, involuting dorsal mesoderm (DM) were dissected and combined to form aggregates according to the method of Wacker et al. (2000). Aggregates were made with treated or untreated BCRs combined with treated or untreated DM pieces. BCRs were cultured on 2% agarose with the inner surface facing up, and two to three DM pieces were placed on this surface. After 40 min of culture, each DM was scored for whether it remained separated as a compact mass in the inner surface of the BCR, or had integrated into the BCR.

### ß-Galactosidase Activity Assays

Chemiluminescent quantification of the reporter pRAREhsplacZ plasmid (Rossant et al., 1991) activity was performed using ß-gal Juice Plus (PJK, Germany) as previously described (Yelin et al., 2005). Chemiluminescence activity was measured on a TD-20/20 Luminometer (Turner Designs). *LacZ* RNA was prepared from a clone containing a nuclear localization signal (pSP6nuc ß-gal) in pGEM-3Z (Promega). The staining of embryos for ß-galactosidase activity was performed with 5-bromo-4-chloro-3-indolyl-β-d-galactopyranoside (Xgal).

### Statistical Analysis

All statistical comparisons were carried out using the Prism software package (Graph Pad Software Inc. San Diego, CA). Results are given as the mean ± standard error of the mean (SEM). Tests used were the 2-tailed *t*-test for two-sample comparisons, Dunnett’s (ANOVA) multiple comparisons test, or Fisher test. Differences between means were considered significant at a significance level of *p* < 0.05.

## Results

### Inhibition of RA Biosynthesis Delays the Progression Through Gastrulation

**FIGURE 1:**
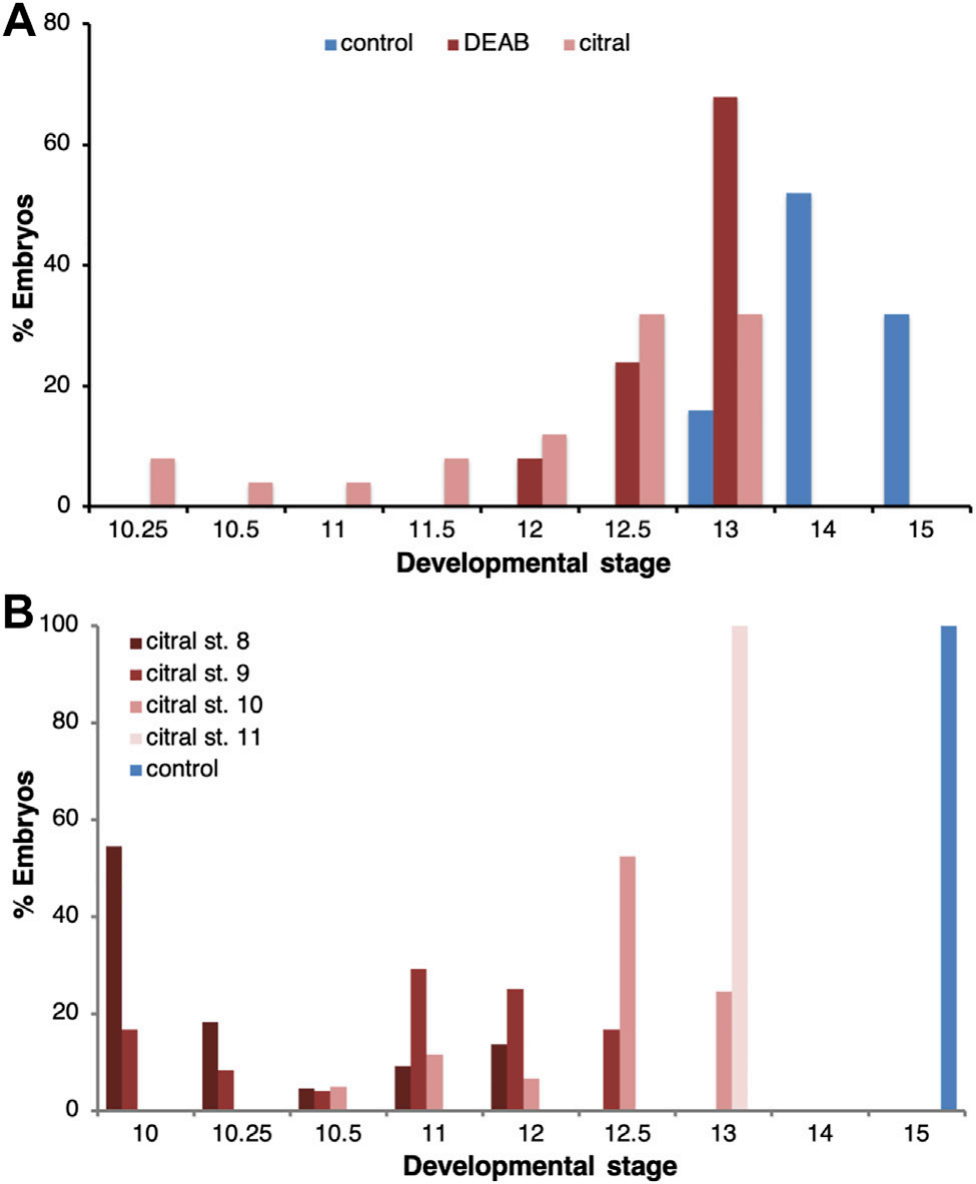
Delayed progression through gastrulation as a result of RA biosynthesis inhibition. **(A)** The progression of embryos through gastrulation treated with DEAB (300 μM; n = 38) or citral (70 μM; n = 39) to inhibit RA biosynthesis. When the majority of control embryos reached early neurula (st. 14/15; n = 38), the stage distribution of the treated samples was determined. The percentage of embryos at each stage in each treatment is shown. **(B)** The temporal sensitivity window for the delay in gastrulation induced by inhibition of RA biosynthesis was studied by initiating the citral treatment at different developmental stages (8, n = 22; 9, n = 24; 10, n = 61; 11, n = 24). When control embryos reached early neurula (st. 15), the stage distribution of the treated samples was determined. The percentage of embryos at each stage in each treatment is shown.

To characterize the effect of reduced RA signaling on gastrulation, we performed a series of experiments employing two different inhibitors of RA biosynthesis, 4-diethylaminobenzaldehyde (DEAB) and 3,7-dimethyl-2,6-octadienal (citral) (Shabtai et al., 2018). Both inhibitors were used at relatively high, but sub-lethal concentrations (DEAB, 300 μM; citral, 70 µM), as previously determined (Shukrun et al., 2019). Groups of embryos were treated with one of the RA biosynthesis inhibitors from the midblastula transition (st. 8.5) (Nieuwkoop and Faber, 1967) and allowed to develop until the majority of the embryos in the control group reached early neurula stages (st. 14) (Figure 1A), at which time both control and treated embryos were fixed and staged. Both RA biosynthesis inhibitors induced a delay in the progression through gastrulation; when sibling control embryos reached stages 13–15, 86.8% of the DEAB-treated group and 76.9% of the citral-treated group were at stages 12–13 (Figure 1A). None of the treated embryos advanced beyond st. 13. These results support previous observations that RA signaling is required for the normal progression through gastrulation (Durston et al., 1989; Sive et al., 1990).

To determine whether there is a critical window for the requirement of RA signaling, RA biosynthesis inhibition (citral) was initiated at different developmental stages and the treatment continued until the control group reached early neurula (st. 15). Analysis of the stage distribution in the experimental groups again showed that RA biosynthesis inhibition delayed gastrulation irrespective of the stage at which the inhibition was initiated (Figure 1B). While most control embryos reached stage 15, when embryos were treated with citral starting at stage 9 they only reached stage 11–12.5 and when the treatment was initiated at stage 11, they only reached stage 13 (Figure 1B). Thus, the earlier the citral treatment was initiated, the more severe the delay in gastrulation. These observations indicate that RA is required for the progression through gastrulation at all stages studied but late blastula/early gastrula appears more sensitive to its inhibition.

### RA Signaling Affects the Migration of the Early Involuting Cells

**FIGURE 2:**
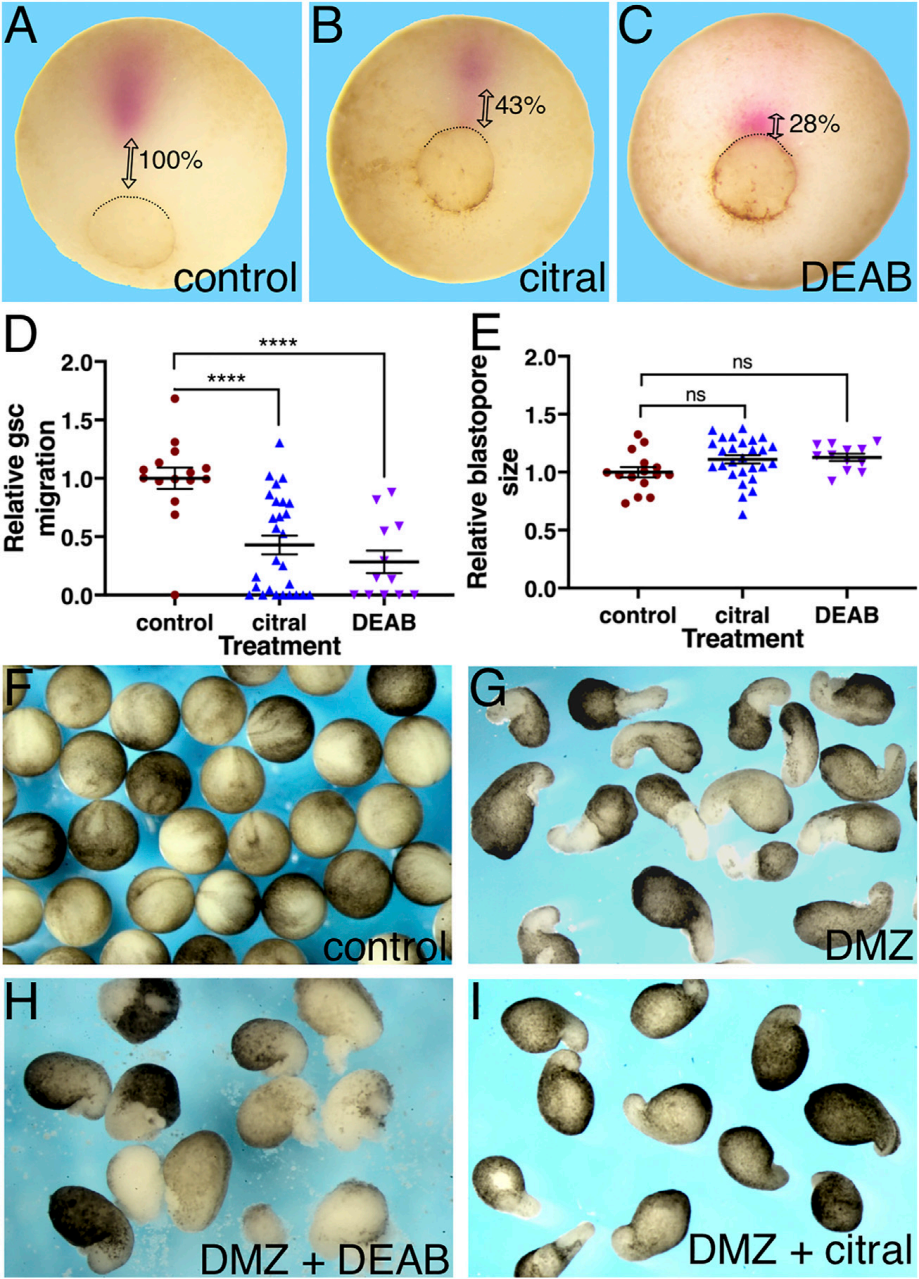
RA inhibition delays the rostral migration of the PCM. **(A–E)** Embryos were treated with citral or DEAB to inhibit the biosynthesis of RA. The extent of migration of the PCM during involution was determined by measuring their distance from the dorsal lip of the blastopore. The PCM cells were identified by *gsc in situ* hybridization. **(A)** Control embryo (n = 15). **(B)** Citral (70 µM) treated embryo (n = 27). **(C)** DEAB (300 µM) treated embryo (n = 12). **(D)** Relative PCM migration distribution. The migration distance was normalized to the embryo diameter. **(E)** Relative blastopore size distribution indicates embryos were at the same developmental stage. For each embryo, we measured its diameter and the diameter of the blastopore and calculated the ratio between them. **(F–H)** The inhibitory effect of reduced RA signaling was studied in explanted DMZs. RA biosynthesis was inhibited in explanted DMZs by incubation in citral or DEAB until control embryos reached st. 18 **(F)**. **(G)** Control DMZ explants all had elongated columns of unpigmented mesoderm (n = 34) whereas in DEAB-treated DMZ explants (n = 16) **(H)** and citral-treated DMZ explants (n = 27) **(I)** these were missing or stunted. ****, *p* < 0.0001; ns, not significant.

A major parameter during *Xenopus* gastrulation that contributes to assigning an embryo to a particular stage is the size of the blastopore, which is a reflection of the extent of involution (Nieuwkoop and Faber, 1967; Keller and Shook, 2008; Keller and Sutherland, 2020). Therefore, we next assessed the effects of RA biosynthesis inhibition on cell involution. Since involution initiates at the dorsal blastopore lip, i.e., the organizer, and is followed by the rostral the migration of the earliest invaginating cells, the LEM/PCM, embryos treated with DEAB or citral were allowed to develop to a late gastrula stage (st. 12) and probed for *gsc* expression by *in situ* hybridization (Cho et al., 1991) to identify the PCM cells (Huang and Winklbauer, 2018). Inhibition of RA biosynthesis by either DEAB or citral resulted in a delay in the rostral migration of the *gsc*-positive cells compared to the control samples (Figures 2A–C). To quantitate the extent of delayed migration, the distance the *gsc*-expressing PCM cells migrated from the blastopore was measured. Migration of the PCM is a very dynamic process dependent on the progression of gastrulation. To minimize the effect of slight size differences between the embryos (Leibovich et al., 2020), the migration distance was normalized to the embryo diameter and presented as a relative migration from the value in control embryos (Figure 2D). The results showed that citral treatment inhibited PCM migration by 57.1% and DEAB inhibited it by 71.7%. This significant migration delay was observed despite the fact that all embryos were allowed to develop to the same developmental stage. During gastrula stages, the size of the blastopore decreases constantly, which normally is linked to the extent of PCM migration (Kaneda and Motoki, 2012; Keller and Shook, 2008). To ensure that our visual staging seleted embryos within a limited range of blastopore sizes, for each embryo we measured its diameter and the diameter of the blastopore and calculated the ratio between them (Figure 2E). The use of this ratio has been shown to normalize staging of embryos of different sizes during gastrulation stages (Leibovich et al., 2020). This comparison of the relative blastopore sizes supported the conclusion that the treated and control embryos were at statistically similar developmental stages, and that the reduction of PCM migration was a direct result of reduced RA signaling rather than overall developmental delay.

To corroborate the RA requirement for the rostral morphogenetic movements of the PCM, we explanted dorsal marginal zones (DMZs) from control and treated embryos. DMZ explants undergo elongation that recapitulates in part the morphogenetic movements of the dorsally involuting cells (Shih and Keller, 1992). The DMZs were incubated until the control sibling embryos reached stage 18 (Figure 2F). In support of the previous conclusion of a delay in the PCM rostral migration (Figure 2D), the DMZ explants treated with either DEAB (Figure 2H) or citral (Figure 2I), exhibited a partial inhibition in their elongation, whereas DMZs explanted from control embryos exhibited the expected elongation resulting from morphogenetic movements of dorsal regions in the *Xenopus* embryo (Figure 2G). These results further support that RA signaling is required for the normal morphogenetic movement of dorsal cells during gastrulation.

We previously showed that the effects on the rostral migration of the PCM by RA signaling reduction with ethanol are transient and these cells reach their normal cranial position by early neurula stages (Yelin et al., 2007). In accord with this finding, analysis of the *gsc* and *chrd.1* expression patterns during early neurula stages (st. 13), revealed that in embryos treated with either DEAB or citral, the PCM cells expressing these markers reached their normal cranial position (Supplementary Figure S1). Interestingly, the notochord, another *chrd.1*-expressing tissue, appears shortened in the embryos with reduced RA levels (Supplementary Figure S1E,F). These observations show that the effect of reduced RA levels on the rostral migration of the PCM cells is transient, while there might be an additional effect on the convergent extension of the notochord (Wilson et al., 1989; Keller and Jansa, 1992; Yelin et al., 2007).

### RA Signaling is Required for Fibronectin Deposition During Gastrulation

**FIGURE 3:**
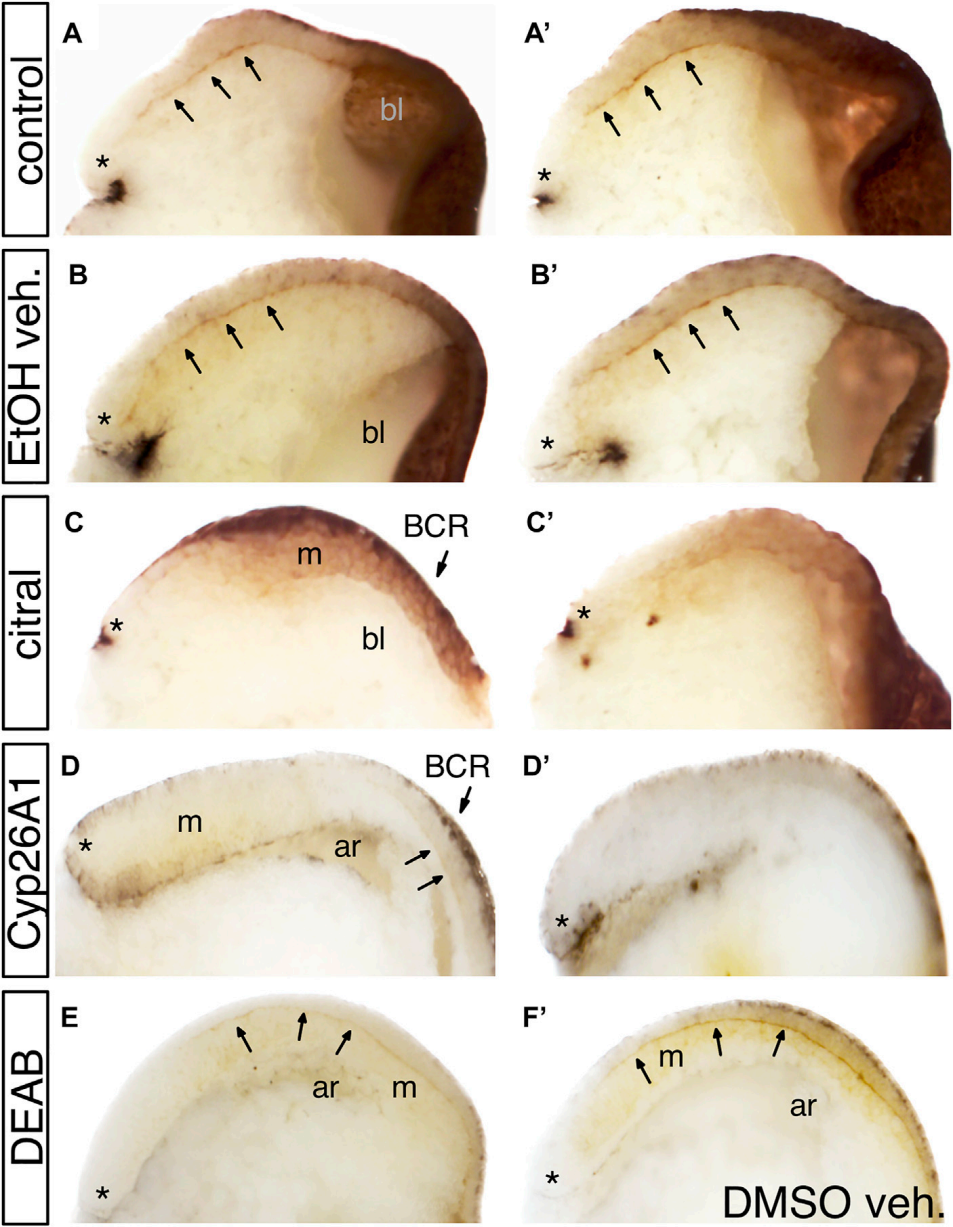
The effect of reduced RA signaling on fibronectin (FN) deposition. Embryos were treated with DEAB or citral or injected with RNA encoding CYP26A1 to reduce RA levels. **(A,A′)** Two examples of untreated sibling control embryos. Arrows point to fibronectin deposited along Brachet’s cleft. * indicates the dorsal lip of the blastopore; bl, blastocoel. **(B,B′)** Two examples of embryos treated with EtOH vehicle only. FN deposition was equivalent to untreated controls. **(C,C′)** Two examples of embryos treated with citral. FN is detected in the ectoderm of the blastocoel roof (BCR) and mesoderm (m), but is not deposited along Brachet’s cleft. **(D,D′)** Two examples of embryos injected with *cyp26a1* mRNA. FN is not detected in the BCR, the mesoderm (m), or Brachet’s cleft (arrows). ar, archenteron. **(E)** Embryo treated with DEAB showed reduced FN staining along Brachet’s cleft compared to a sibling embryo treated only with DMSO vehicle **(F)**.

Previous studies showed that mesoderm involution requires the extracellular deposition of fibronectin (FN) along Brachet’s cleft (Winklbauer and Schürfeld, 1999; Gorny and Steinbeisser, 2012). To assess whether the disrupted involution and rostral migration of the PCM cells after inhibition of RA biosynthesis might involve loss of FN deposition, we analyzed early/mid gastrula (st. 10.5) embryos by immunostaining with anti-FN antibodies (Davidson et al., 2004). RA levels were reduced by either citral or DEAB treatment, or by injection of RNA encoding CYP26A1, an enzyme belonging to the cytochrome P450 family that renders RA biologically inactive and targets it for degradation (Ribes et al., 2007; Thatcher and Isoherranen, 2009). We found that reducing RA levels by these three different methods reduced FN deposition along Brachet’s cleft during gastrulation. In control embryos, a thin line of FN deposition along Brachet’s cleft is notable in stage 10.5 gastrula embryos (Figure 3A, A’; n = 47). The same FN staining was observed in vehicle-treated siblings (EtOH: Figure 3B, B′, n = 20; DMSO: Figure 3F, n = 24). However, embryos treated with either citral (n = 15) or DEAB (n = 52) to block RA signaling (Figure 3C, C′, E), or embryos overexpressing the RA hydroxylating enzyme CYP26A1 (Figure 3D, D′, n = 18) showed severely reduced to not detectable FN-immunostaining in Brachet’s cleft. In samples showing reduced FN staining, the cytoplasmic FN in both the ectodermal BCR and the MZ mesoderm was reduced, indicating that the defect was not specific to one germ layer (e.g., Figure 3 C, C′). These assays indicate that a major target of disrupted RA signaling during gastrulation is the deposition of a suitable extracellular FN matrix upon which the LEM/PCM cells can migrate along the BCR in a rostral direction.

### Reduced RA Biosynthesis Results in Abnormal Gastrula Morphogenetic Movements

**FIGURE 4:**
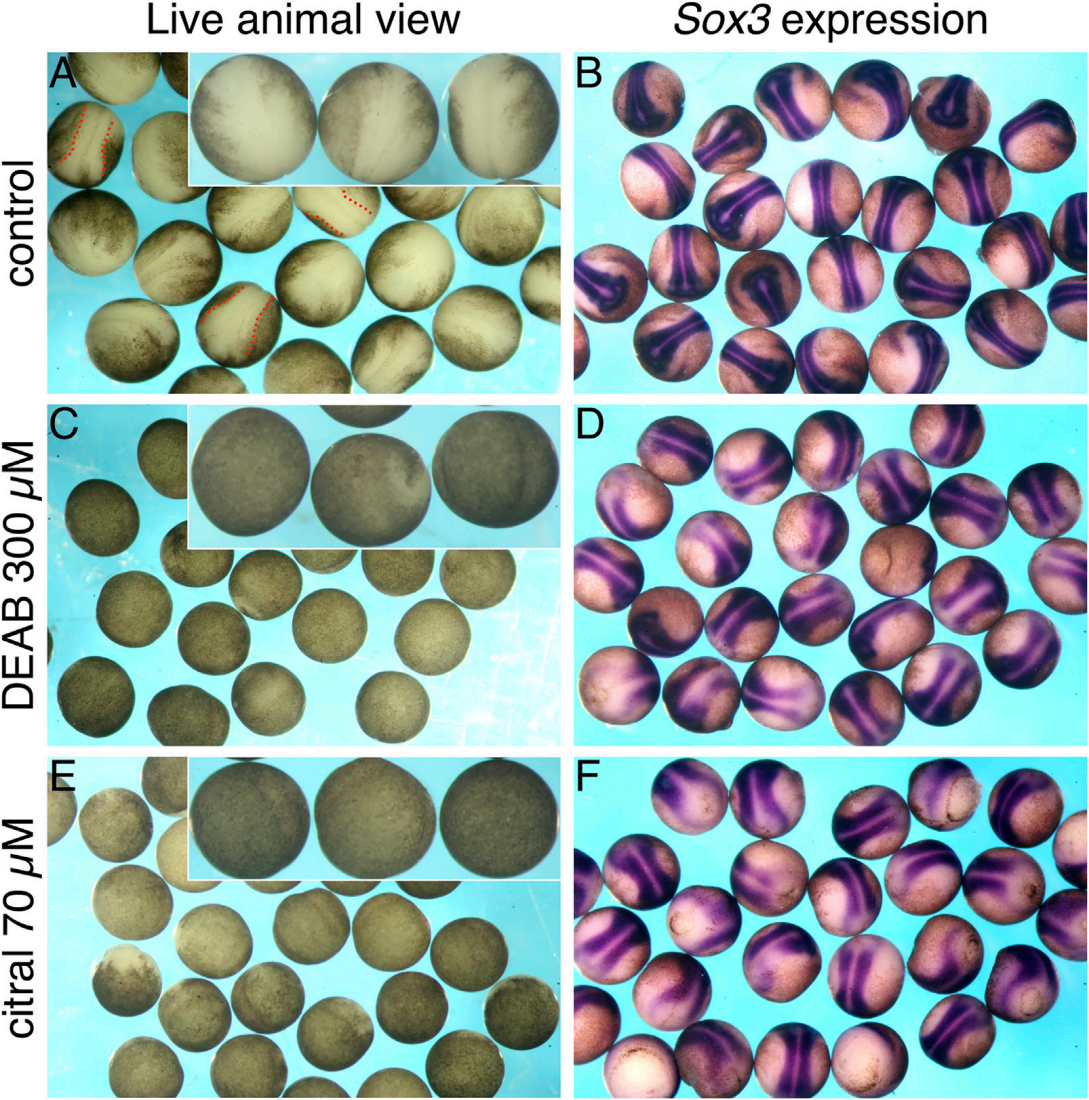
Reduced RA signaling affects the apparent location of the neural plate. Embryos were treated with DEAB or citral to inhibit the biosynthesis of RA. At st. 15, embryos were analyzed for the formation of the neural plate while alive by visual examination **(A,C,E)**, or after *in situ* hybridization with the neural plate marker, *sox3* **(B,D,F)**. **(A,B)** Control embryos (n = 25). **(C,D)** Embryos treated with DEAB (300 μM; n = 23). **(E,F)** Treatment of embryos with citral (70 μM; n = 26).

Incubation of embryos treated with DEAB or citral to early neurula stages (st. 15) uncovered another morphogenetic defect from the efficient inhibition of RA signaling. When observing living control embryos, their natural buoyancy in the aqueous medium causes the dorsal side containing the neural plate to face up in the culture dish (Figure 4A). In contrast, living DEAB- or citral-treated embryos in the culture dish appeared to lack a neural plate (Figures 4C,E). However, manually turning the living DEAB- or citral-treated embryos revealed the presence of an apparent neural plate on the side of the embryo facing the dish, which normally would be the ventral side. Processing these embryos for *in situ* hybridization with the neural plate marker, *sox3* (Penzel et al., 1997), confirmed the presence of neural plate tissue (cf. Figure 4B to Figures 4D,F). These observations suggested that reduction of RA signaling caused the neural plate to form on the ventral side of the embryo.

**FIGURE 5:**
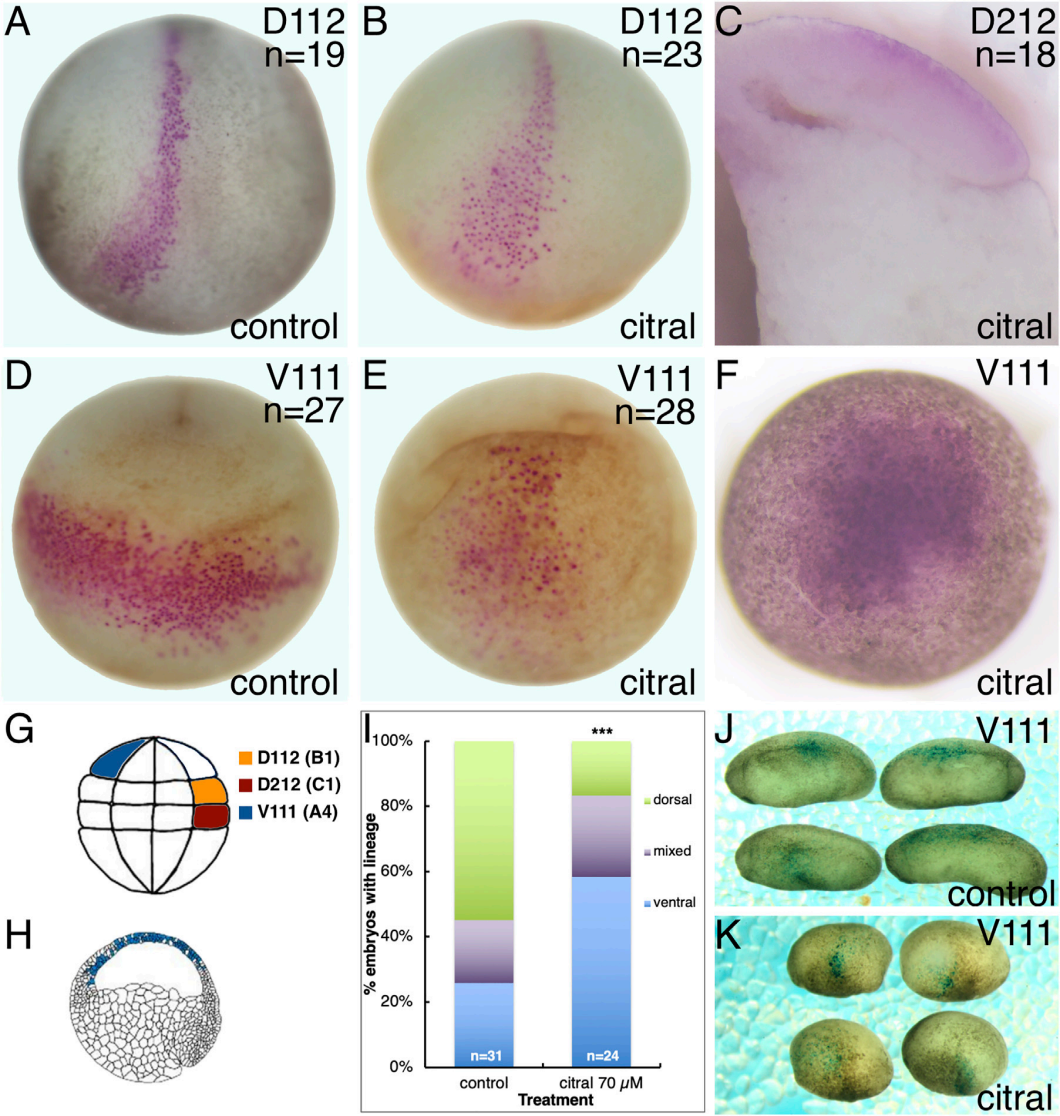
Reduced RA signaling does not alter the dorsal-ventral axis. Single 32-cell blastomeres were injected with lineage tracer mRNA and labeled clones mapped at different developmental stages. **(A,B)** At stage 15, the D112/B1 clone (pink cells) occupies the medial and anterior neural plate in both untreated control **(A)** and citral treated **(B)** embryos. Dorso-anterior views. **(C)** The D212/C1 blastomere that normally contributes to the involuting dorsal mesoderm at gastrulation (st 10.5; Bauer et al., 1984) also does so in citral-treated embryos. Mid-sagittal section with anterior to the left and dorsal to the top. **(D)** At stage 17, the V111/A4 clone in untreated control embryos forms a coherent ventrolateral clone. Ventral view with anterior to the top. **(E)** In citral-treated sibling embryos, the stage 17 V111/A4 clone remains medial, i.e., has not expanded laterally, and the cells are more dispersed. Ventral view with anterior to the top. **(F)** At gastrula stages (st 10.5), the V111/A4 clone in citral-treated embryos occupies the blastocoel roof, similar to control embryos (cf. H). **(G)** Location of the 32-cell blastomeres that were lineage traced. Dorsal to the right, animal to the top. **(H)** Location of the V111/A4 clone (blue cells) at early gastrulation, modified from Bauer et al. (1984). Animal to the top and dorsal to the right. **(I)** Percentage of embryos in which the V111/A4 clone was located predominantly in the dorsal, ventral, or both (mixed) epidermis at tailbud stages. **(J)** Examples of control embryos showing V111/A4 descendants (blue cells) mostly in the dorsolateral epidermis. Dorsal to the top, anterior to the left. **(K)** Examples of citral treated embryos showing V111/A4 descendants (blue cells) mostly in the ventral and ventrolateral epidermis. Dorsal to the top, anterior to the left.

We tested the possibility of respecification of cell fates by lineage tracing the blastomere progenitors of the neural plate (Figure 5). *Xenopu*s blastomeres with different fates can be visually distinguished by the location of the first cleavage furrow and darker pigmentation of the ventral-animal quadrant (Klein, 1987; Moody, 2018). Therefore, we lineage labeled a single dorsal-animal blastomere of the 32-cell embryo (D112) (Jacobson and Hirose, 1981; Moody, 1987), also known as B1 (Nakamura et al., 1978; Dale and Slack, 1987) (Figure 5G) that is the major progenitor of the neural plate (Dale and Slack, 1987; Moody, 1987) to mark its progeny at later stages. In sibling controls, the D112 clone was located in the neural plate extending along the dorsal midline from the posterior blastopore to a broadened fan in the anterior neural plate (Figure 5A), in accord with published fate maps (Moody, 1987; Dale and Slack, 1987). The D112 clone in every citral-treated neurula embryo was distributed in an identical pattern (Figure 5B), indicating that reduced RA signaling does not cause a reversal in cell fate maps. This is supported by the observation that in the gastrula (st. 10.5), the labeled clone of cells derived from a dorsal-vegetal blastomere (D212, aka C1; Figure 5G) that is a major contributor to the involuting organizer mesoderm (Bauer et al., 1994), is located in the dorsal lip in citral-treated embryos (Figure 5C). Thus, blastomeres that normally contribute to dorsal ectoderm (D112) and dorsal mesoderm (D212) continue to do so in embryos in which RA signaling was reduced; the cell fate map is not altered.

However, the distribution of ventral ectodermal clones was altered by reduced RA signaling. Labeling a single ventral-animal blastomere of the 32-cell embryo (V111, aka A4; Figure 5G) produces a coherent clone stretching across the ventrolateral epidermis of the neurula (Figure 5D), as previously described (Dale and Slack, 1987; Moody, 1987). Although the V111 clone in citral-treated neurulae also was located in the ventral epidermis, it was more midline and the cells were more dispersed (Figure 5E). Since the V111 clone is confined to the animal cap ectoderm at gastrula stages (10.5), as expected for prospective epidermal cells (Figures 5F,H), these results confirm that the dorsal-ventral fate map is not reversed, but that there is an interruption or delay in morphogenetic movements. Performing the lineage analysis of the V111 (A4) blastomere at tailbud stages (st. 30) confirmed this. While most of the control embryos exhibited descendants flanking the neural tube in the dorsolateral epidermis (Figures 5I,J), in the citral-treated embryos this clone was significantly more frequently located in a more ventral position (Figures 5I,K). Perhaps this accounts for the neural plates of the RA-reduced embryos often appearing broader than those of controls (Figures 4B,D,F). Thus, lineage analyses at gastrula, neurula, and tailbud stages demonstrate that reduced RA signaling does not reverse the dorsal-ventral fate map of the embryo, but instead alters some morphogenetic movements that cause living embryos to orient “upside-down” in the culture dish (Figure 4).

### RA Regulates the Internal Reorganization of the Gastrula Embryo

A notable phenotype of the living neural plate stage embryos in which RA signaling was reduced was the presence of a large, fluid-filled cavity oriented in the “up” position facing away from the culture dish (Figures 4C,E)—a position normally occupied by the neural plate (Figure 4A). In control embryos, the blastocoel forms during cleavage stages separating the animal cells from vegetal cells by blastula stages. During gastrulation, the blastocoel is pushed ventrally by the involuting dorsal mesoderm, shrinks in size, and eventually disappears by the end of gastrulation (around st. 13). We hypothesized that the unusual cavity in the RA-disrupted embryo, which causes the ventral side of the embryo to be buoyant so that their ventral side faces “up” (Figures 4C,E), is a persistent blastocoel that is not eliminated due to the abnormal timing and extent of LEM/PCM involution (Figures 1, 2).

**FIGURE 6:**
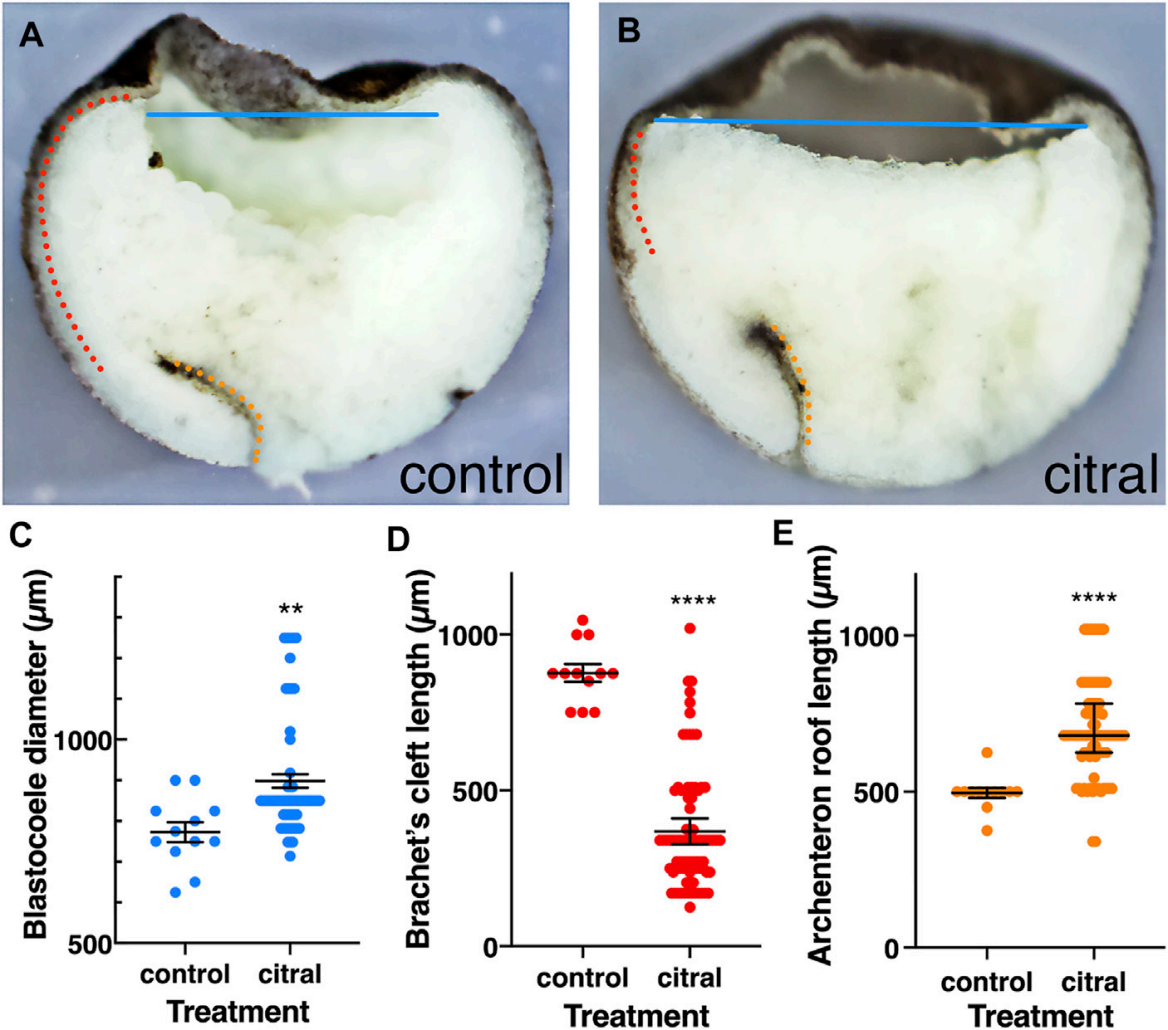
Internal reorganization of the embryo as a result of reduced RA signaling. Control (n = 12) and citral-treated (n = 84) embryos were incubated to mid-gastrula (st. 11) and bisected sagittally. **(A,B)** Control and citral-treated bisected embryos. Blastocoele diameter, blue line; Brachet’s cleft; dotted red line; Archenteron roof, dotted orange line. **(C)** Distribution of the blastocoel diameter in control and citral-treated embryos. **(D)** Measurement of Brachet’s cleft length in citral-treated and control embryos. **(E)** Length of the archenteron roof in citral-treated and control embryos. Citral-treated embryos were compared to the control values. **, *p* < 0.01; ****, *p* < 0.0001.

Therefore, we examined the internal morphology of gastrulating citral-treated embryos. In control embryos that reached mid-gastrula (st. 11), the LEM/PCM have moved along the BCR, compressing the blastocoel into an ovoid shape (Figure 6A). In contrast, although the dorsal lip of the blastopore forms in citral-treated siblings that were time-matched to controls, the blastocoel remains wide with a flat floor that extends across the entire animal hemisphere (Figure 6B). Indeed, measuring the blastocoel diameter in time-matched untreated embryos and their citral-treated siblings confirmed that the blastocoel is significantly larger in citral-treated embryos (Figure 6C). Another morphological change indicating a disruption of mesoderm involution is the significantly shorter length of Brachet’s cleft. This cleft, located between the involuting dorsal mesoderm and BCR is created by the BCR pushing down on the mesoderm and the vegetal endoderm rotating to push the leading edge of the mesoderm toward the animal pole (Winklbauer and Schürfeld, 1999; Yanagi et al., 2015). Brachet’s cleft is significantly shorter in citral-treated embryos compared to untreated time-matched siblings (Figures 6B,D). This is not due to a failure of the archenteron to form (Figure 6B). In fact, the length of the archenteron was significantly longer in citral-treated embryos (Figure 6E), which likely is due to the ability of the vegetal cells to internalize and blastopore closure to take place in the absence of mesoderm involution, as shown in embryos after the removal of the blastocoel roof (Keller and Jansa (1992). Together, these morphological measurements indicate that one consequence of reducing RA signaling by citral treatment is the disruption of the progression of LEM/PCM involution during gastrulation.

### RA is Required for Efficient Tissue Separation and Wnt/PCP Signaling

**FIGURE 7:**
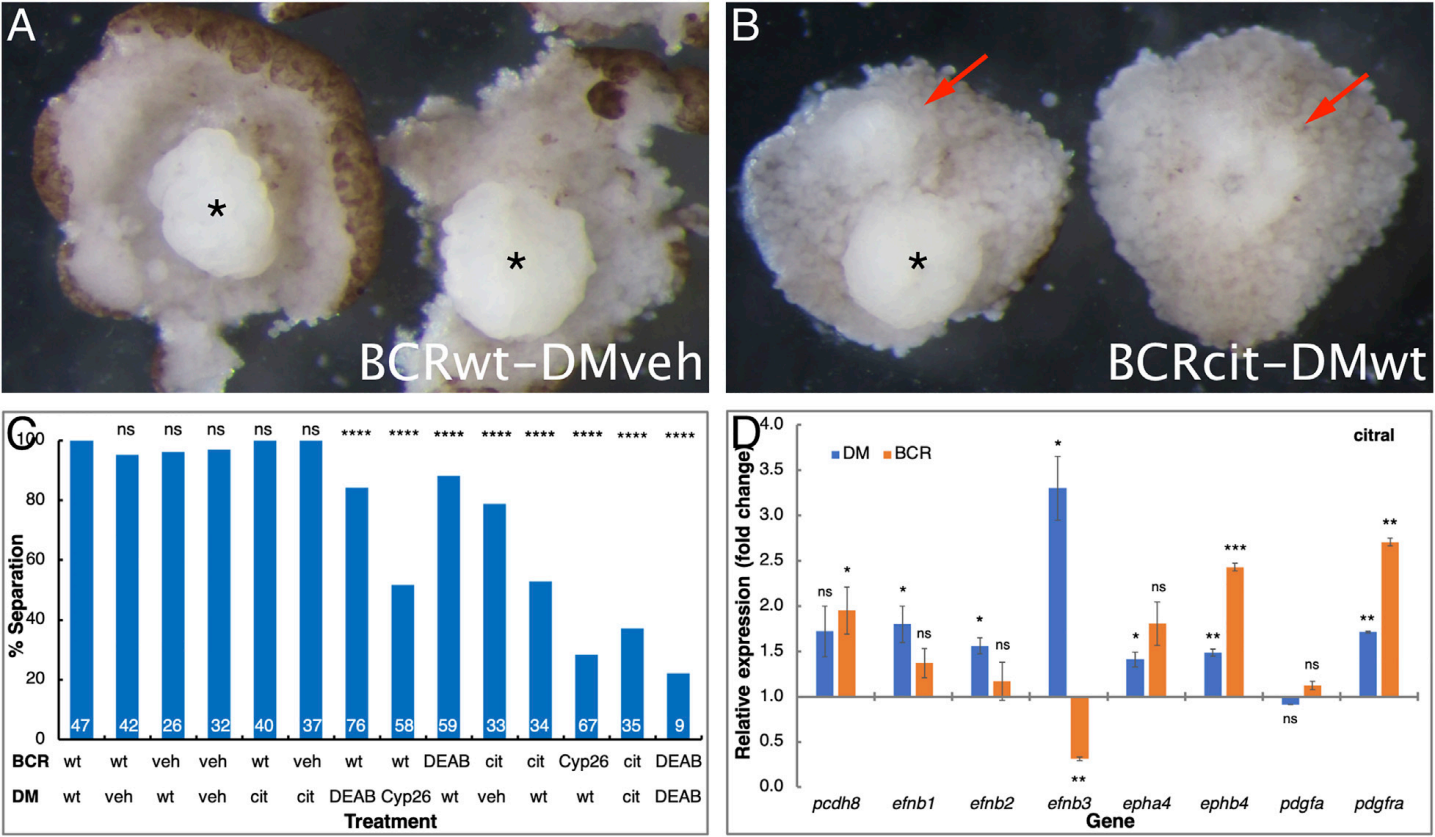
RA regulates the tissue separation behavior across Brachet’s cleft. Embryos were manipulated to reduce RA signaling levels by treatment with DEAB, citral, or injected with mRNA encoding CYP26A1. For controls, embryos were treated with the diluent alone (veh) for the DEAB or citral dilutions or left untreated (wt). The explanted BCR or DM regions were analyzed separately or conjugated for the separation behavior assay. **(A)** Control examples of separation behavior assay conjugates between control (wt) BCR and vehicle (veh) treated DM (asterisk). **(B)** Loss of separation behavior (arrows) when control DM was placed onto BCR treated with citral. * indicates a DM that did remain separated. **(C)** The extent of separation behavior (%) in all the assays performed in all combinations. # separation/# total: 47/47 DMwt/BCRwt; 40/42 DMveh/BCRwt; 25/26 DMwt/BCRveh; 31/32 DMveh/BCRveh; 40/40 DMcit/BCRwt; 37/37 DMcit/BCRveh; 64/76 DMDEAB/BCRwt; 30/58 DMCyp26/BCRwt; 52/59 DMwt/BCRDEAB; 26/33 DMveh/BCRcit; 18/34 Dmwt/BCRcit; 19/67 DMwt/BCRCyp26; 13/35 DMcit/BCRcit; 2/9 DMDEAB/BCRDEAB. **(D)** qPCR analysis of *pcdh8*, *efnb1*, *efnb2*, *efnb3*, *epha4*, *ephb4*, *pdgfa*, and *pdgfra* gene expression changes in the DM and BCR following citral treatment. **, *p* < 0.01; ***, *p* < 0.001; ****, *p* < 0.0001; ns, not significant.

Previous studies showed that the movement of the LEM/PCM upon the blastocoel roof requires a repulsive interaction between these two populations across Brachet’s cleft, termed tissue separation (Wacker et al., 2000; Winklbauer et al., 2001). To determine whether the migratory deficiency in RA-disrupted embryos originates from either the cells in the dorsal mesoderm (DM) or the blastocoel roof (BCR), we performed a separation behavior explant assay, designed to test the regulation of tissue separation across Brachet’s cleft (Winklbauer et al., 2001). We tested whether the RA-disrupting treatments specifically affected either the DM or BCR by hampering the separation behavior required for invagination and rostral migration (Gorny and Steinbeisser, 2012). Explants composed of DM and BCR from RA-reduced treated and control samples in multiple combinations were analyzed. As previously shown (Winklbauer et al., 2001; Gorny and Steinbeisser, 2012), when untreated DM (DMwt) were explanted onto vehicle-treated BCR (BCRveh), or when the reverse combination was performed, the explants remained separated in nearly every case (Figures 7A,C). The regulatory role of RA signaling in tissue separation across Brachet’s cleft then was analyzed in explants treated with either DEAB, citral, or overexpressing CYP26A1. RA signaling reduction in the BCR consistently and robustly hindered the separation behavior resulting in the sinking of the DM into the BCR (Figures 7B,C). Interestingly, RA signaling reduction in the dorsal mesoderm also hindered the separation behavior, although it had a weaker effect (Figure 7C). RA manipulation of both explants prior to performing the separation assay resulted in an additive effect supporting the role of RA in both the DM and the BCR for the separation behavior (Figure 7C). The separation behavior is clearly affected by RA signaling acting on both the DM and the BCR. This is consistent with reduced FN expression in both the ectoderm and mesoderm (Figure 3C, C′).

Previous studies identified a number of molecules that are responsible for the formation of Brachet’s cleft, tissue separation, and LEM involution (reviewed in Gorny and Steinbeisser, 2012). To identify whether any of the genes encoding these proteins are perturbed by disrupting RA signaling during gastrula, we dissected the DM and BCR from citral and vehicle-treated embryos and their untreated siblings. RNA from DM, BCR, and whole embryos was analyzed by qPCR (Figure 7D). Analysis of *pcdh8*, *efnb1*, *efnb2*, *efnb3*, *epha4*, *ephb4*, *pdgfa*, and *pdgfra*, revealed that RA signaling fulfills a complex regulatory role in the interaction between the DM and the BCR. In the DM, the citral treatment resulted in significant up-regulation of the *efnb1*, *efnb2*, *epha4*, *ephb4*, and *pdgfra* genes suggesting that RA negatively regulates their expression (Figure 7D). The citral treatment also significantly up-regulated the expression of *ephb4* and *pdgfra* in the BCR, again supporting a fine-tuning role of RA along Brachet’s cleft. Only the expression of *efnb3* was significantly down-regulated by citral in the BCR, suggesting that normal RA signaling is required for the expression of this gene in the non-involuting ectoderm. The reduction in *efnb3* expression is also in agreement with the observation that any RA reduction in the BCR hampers the tissue separation behavior across Brachet’s cleft (Figures 7A–C). These results also demonstrate that reduced RA signaling affects both the DM and BCR.

**FIGURE 8:**
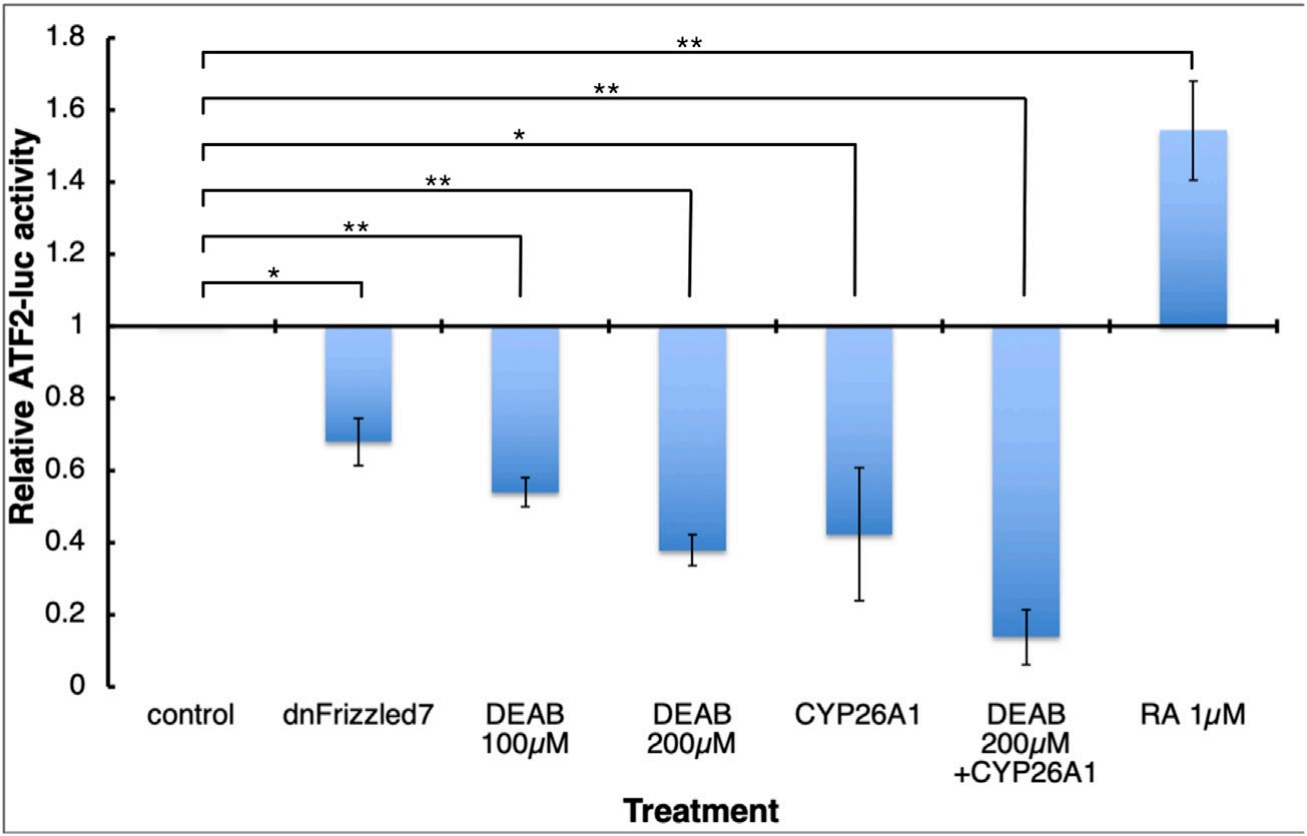
RA signaling is necessary for normal Wnt/PCP activity. Embryos were injected with the ATF2-based non-canonical Wnt signaling reporter plasmid and subjected to DEAB or RA treatments, or co-injected with mRNA encoding dominant-negative Frizzled 7 or CYP26A1. During late gastrula (st. 12) embryos were collected and analyzed and the effect on the reporter activity (luciferase) was determined. *, *p* < 0.05; **, *p* < 0.01.

Previously, Frizzled7-dependent non-canonical Wnt signaling was shown to be important for the tissue separation behavior (Winklbauer et al., 2001; Medina et al., 2004; Luu et al., 2015). Although Wnt/PCP signaling can proceed through several downstream signaling pathways (Sokol, 2015; Ng et al., 2019), the ATF2-based non-canonical Wnt signaling reporter plasmid (Ohkawara and Niehrs, 2011) allows us study the effect of RA signaling manipulation on the non-canonical Wnt activity. Embryos were injected along the marginal zone with the ATF2 reporter plasmid and either co-injected with mRNA encoding CYP26A1 or treated with DEAB. During late gastrula (st. 12) embryos were collected, and the level of luciferase activity was determined. The activity of the ATF2 reporter plasmid exhibited a concentration-dependent reduction in response to increasing DEAB concentrations (Figure 8). A similar reduction was observed in embryos overexpressing CYP26A1 and this effect was enhanced by adding DEAB to these embryos. In agreement with a promoting effect of RA signaling on non-canonical Wnt signaling, treatment with RA increased the transcriptional activity of the reporter plasmid (Figure 8). As a control for the involvement of non-canonical Wnt signaling in the activity of the ATF2 reporter, RNA encoding a dominant-negative form of the Frizzled 7 receptor was also co-injected resulting in a significant reduction as expected. These results support a requirement for RA signaling for the non-canonical signaling activity during gastrulation.

## Discussion

### Reduced RA Delays Gastrulation and Morphogenetic Movements

RA signaling is well established to play pivotal roles in embryonic axis formation and patterning. Supplying excess RA to the culture medium affects progression through *Xenopus* gastrulation, including a delay in the closure of the blastopore (Durston et al., 1989; Sive et al., 1990). Excess RA signaling caused by DHRS3 knockdown, a retinaldehyde reductase whose normal activity reduces the production of RA, affects the migration of the *gsc*- and *lhx1*-positive cells during gastrulation (Kam et al., 2013). Other studies have relied on pharmacological inhibitors of RA biosynthesis to reduce endogenous RA levels and shown similar effects, as discussed below. The similar effects of increased and decreased RA signaling on the progression of gastrulation suggests a complex regulatory role of RA during early embryogenesis (Gur et al., 2022).

The two most commonly used pharmacological inhibitors of RA biosynthesis are DEAB and citral. Our studies using DEAB or citral concentrations above the inhibition constants (*K*_*i*_) for the human ALDH1A2 enzyme (Shabtai et al., 2016) revealed an as yet uncharacterized effect of reduced RA signaling: a significant delay in the progression through gastrulation. Similar results were observed when embryos were treated with ethanol, another RA biosynthesis inhibitor (Shabtai et al., 2018; Yelin et al., 2007, 2005). Two retinaldehyde dehydrogenases that produce RA, ALDH1A2*,* and ALDH1A3, are expressed during early gastrulation (Chen et al., 2001; Blentic et al., 2003; Halilagic et al., 2003; Lupo et al., 2005; Shabtai et al., 2018; Gur et al., 2022). Mutation of each of these genes in vertebrate embryos results in post-gastrula delays and loss of viability (Niederreither et al., 1999; Begemann et al., 2001; Sandell et al., 2007). Therefore, we propose that the pharmacological inhibitors of RA biosynthesis used in these and the present study (DEAB, citral, ethanol) likely target more than one ALDH enzyme during early embryogenesis (Morgan et al., 2015; Cho et al., 2021). The specificity of the RA reduction by these reagents is supported by the similar results obtained by overexpressing Cyp26a1, an RA catabolic enzyme (Yelin et al., 2005; this work).

Reduced RA signaling might delay gastrulation movements by a number of mechanisms. Although germ layer induction or patterning could be affected, we observed the appropriate dorsal midline expression of *gsc*-, and *chrd.1*-positive cells. In other studies, reduced RA signaling by ethanol treatment or Cyp26a1 overexpression also showed dorsal midline expression of *gsc-*, *chrd.1*-, *otx2*-, and *not*-positive cells (Yelin et al., 2007, 2005). Thus, prechordal mesoderm appears to be induced and appropriately patterned. However, subtle effects on expression levels on these genes and other components of the mesoderm network should be further studied.

One consistent effect of these various methods of reducing RA, however, was a delay in the movement of *gsc*-positive PCM cells from the organizer to their ultimate cranial position, which in normal embryos results in the formation of the notochord (De Robertis et al., 1994; Huang and Winklbauer, 2018). This movement involves convergence-extension movements, which can be assessed by the extent that DMZ explants elongate (Keller and Shook, 2008; Keller and Sutherland, 2020; Winklbauer, 2020). This report and previous studies (Yelin et al., 2005) clearly demonstrate that the elongation of DMZ explants subjected to reduced RA signaling is severely impaired, indicating a reduced capacity of these cells to perform convergence-extension. Interestingly, we previously showed that the *gsc*-positive PCM cells in ethanol treated embryos eventually reach their cranial position (Yelin et al., 2007, 2005), but the delay likely affected inductive interactions with the neural ectoderm that should have taken place earlier (Gur et al., 2022). Together, these reports indicate that in addition to its early role in the organizer RA also has an early effect on the morphogenetic movements of the involuting mesoderm. It will be important to determine whether altered RA signaling affects all subdivisions of mesoderm or specifically LEM, PCM or mesendodermal cells.

### Reduced RA Leads to an Internal Reorganization of the *Xenopus* Embryo

The delayed rostral migration of the cells expressing organizer-specific genes at the onset of gastrulation is probably one of the earliest effects of reduced RA signaling, one consequence of which might be altered patterning of the body axis due to delayed or defective inductive signaling, as previously suggested (Gur et al., 2022). This possibility seemed likely because reduced RA signaling gave rise to embryos whose neural plates appeared to be mislocalized to the ventral side. We performed a series of lineage tracing experiments to determine whether this phenotype resulted from altered cell fates leading to reversed dorsal-ventral axes or abnormal cell movements. For the lineages studied that are destined to give rise to dorsal ectoderm or dorsal mesoderm, the clones exhibited the expected distribution at neurula stages, even under reduced RA signaling conditions. However, the V111 (A4) blastomere, which is fated to give rise to the ventral-lateral epidermis, exhibited an abnormal distribution in embryos subjected to reduced RA signaling. These results ruled out changes in cell fates but supported effects on morphogenetic movements, leading us to postulate that reducing RA signaling at blastula stages would result in an altered internal organization of the embryo.

Consistent with this idea, we noted that reduced RA signaling neurulae appeared to contain a persistent blastocoel that forced the apparent repositioning of the neural plate. Analysis of the blastocoel during gastrula stages in fact demonstrated it to be significantly broader than in controls, indicating a defect in the internalization and subsequent anterior migration of the MZ that is instrumental for blastocoel compaction. We further observed that Brachet’s cleft, an indicator of the initiation of mesoderm migration onto the blastocoel roof, was significantly shorter in reduced RA signaling embryos. LEM/PCM involution and migration requires the deposition of a FN substrate along Brachet’s cleft (Keller and Jansa 1992; Winklbauer and Schürfeld, 1999; Gorny and Steinbeisser, 2012; Huang and Winklbauer 2018; Forecki et al., 2018). We found that reducing RA signaling by three different approaches resulted in a significant reduction in FN deposition, indicating an effect on LEM/PCM migration. Both phenotypes, the shortened Brachet’s cleft and the inefficient deposition of FN, are linked to defective involution of the mesendoderm (Keller and Jansa, 1992; Shook and Keller, 2008; Gorny and Steinbeisser, 2012; Barua et al., 2021). Interestingly, even in the absence of dorsal mesoderm migration, notochord formation anomalies develop, and involution and closure of the blastopore, continues resulting in internalization of the vegetal cells (Keller and Jansa, 1992). In future experiments, it will be important to determine the precise cellular effects of reduced RA signaling on both the migrating mesoderm and the ectodermal substrate utilizing the many sophisticated explant approaches that have been developed in *Xenopus*.

### Inhibition of RA Signaling Affects the Tissue Separation Behavior

The delay in the involution of cells expressing organizer-specific genes (Yelin et al., 2005, 2007; this work), the shortening of Brachet’s cleft and the reduced deposition of FN in the cleft prompted us to study the tissue separation behavior of the involuting marginal zone cells (Gorny and Steinbeisser, 2012; Winklbauer, 2020). Inhibition of RA signaling in either the BCR or the DM cells reduced the extent of tissue separation. The slightly higher sensitivity in the BCR may account for the reduced FN deposition along the cleft, which should be assessed in future experiments. We suggest that defective tissue separation likely is the cause of the shortened Brachet’s cleft due to partial fusion of the mesoderm and ectoderm. However, differences in the relative positions of PCM, chordal mesoderm and mesendoderm in RA inhibitor-treated and control gastrulae might contribute to the observed tissue separation defects. Performing these experiments with precisely fate-mapped pre-involution mesoderm pieces would discriminate between these possibilities.

Nonetheless, the tissue separation defect was shown to most likely involve the non-canonical Wnt signaling pathway as the activity of a non-canonical Wnt signaling reporter plasmid (Ohkawara and Niehrs, 2011) was reduced by inhibiting RA biosynthesis in a concentration-dependent manner. The effect of reduced RA signaling on the non-canonical Wnt pathway phenocopied the known effect of dominant negative Frizzled 7 overexpression on this pathway and on the tissue separation behavior (Winklbauer et al., 2001). Tissue separation also has been shown to also involve multiple ephrins and their Eph receptors in addition to PDGF and paraxial protocadherin (Gorny and Steinbeisser, 2012; Fagotto, 2020). Manipulation of RA levels in the BCR cells, in particular, resulted in the reduction in the expression of *efnb3*. This response suggests that RA signaling is important for the expression of *efnb3* in the ectoderm where it is normally enriched (Fagotto et al., 2014). Many of the other genes tested exhibited some degree of up-regulation following RA knockdown. These observations support the hypothesis that RA signaling plays a regulatory role in the tissue separation behavior during gastrulation.

A number of reports showed a regulatory link between RA signaling and Wnt/PCP, an important pathway regulating multiple morphogenetic movements (Wallingford, 2012; Sokol, 2015; Brinkmann et al., 2016). For example, RA regulates components of the Wnt pathway (Zhao and Duester, 2009; Carron and Shi, 2016) and RA target genes interact with components of this pathway (Harada et al., 2007; Zhang et al., 2013). Ethanol similarly interacts with the Wnt/PCP pathway (Sarmah et al., 2020; Sidik et al., 2021). These observations suggest that the RA effects on gastrulation and the rostral movement of the LEM/PCM involve an interaction with the Wnt/PCP pathway to regulate morphogenetic movements.

## Conclusion

Herein we report that reducing endogenous levels of RA at blastula stages results in multiple defects in the ability of the mesoderm to involute that leads to later morphological defects including a persistent blastocoel, delayed cranial elongation of the notochord and broader neural plate. Together, these observations indicate that one of the earliest roles of RA signaling in the embryo is the regulation of several morphogenetic processes that are critically important for the normal progression of gastrulation. We speculate that an underlying result of delaying the involution and rostral movement of LEM/PCM cells is that some of the ectodermal cells are likely to have lost their competence to respond to mesoderm derived signals. Support for this abnormal inductive timing was observed in embryos manipulated for reduced RA signaling that developed microcephaly (Gur et al., 2022). The remarkable versatility of *Xenopus* explants, including precise dissection of the different mesodermal precursor populations, tissue separation and migration assays, will be instrumental to further characterize the signaling pathways, cell adhesion molecules and substrates that are altered by reduced RA signaling.

## Data Availability

The original contributions presented in the study are included in the article and Supplementary Material, further inquiries can be directed to the corresponding authors.

## Abbreviations

RA, retinoic acid; LEM, leading edge mesendoderm; PCM, prechordal mesoderm; ALDH, aldehyde dehydrogenase; BCR, blastocoel roof; MZ, marginal zone; DMZ, dorsal marginal zone; DM, dorsal mesoderm; MBSH, Modified Barth’s Solution Hepes DEAB, 4-Diethylaminobenzaldehyde; 3,7-Dimethyl-2,6-octadienal, citral; ethanol, EtOH; MBT, mid-blastula transition; veh, vehicle; qPCR, quantitative reverse transcription real-time PCR; FN, fibronectin.
