# Supplementary material for: Retinoic Acid is Required for Normal Morphogenetic Movements During Gastrulation
Source: Front Cell Dev Biol. 2022 Apr 21;10:857230. doi: 10.3389/fcell.2022.857230 (PMC9068879; doi:10.3389/fcell.2022.857230)
Supplement: Supplementary file 1 [file DataSheet1.PDF]

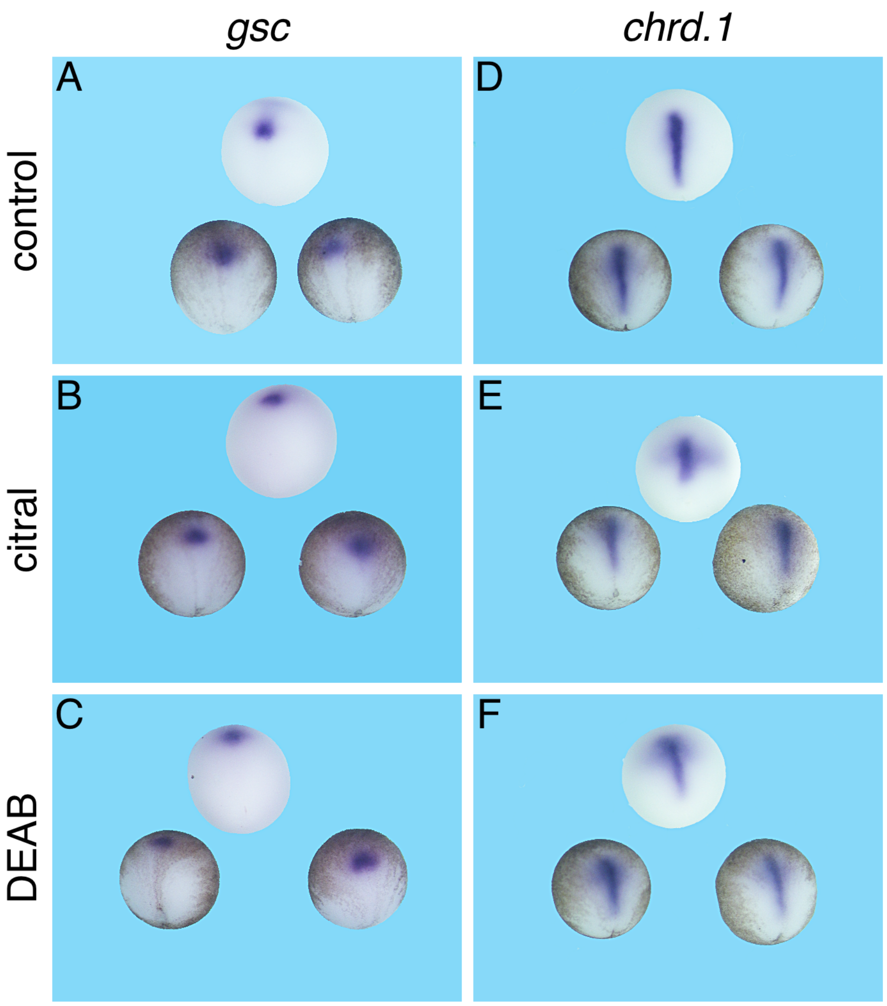

Supplemental Figure S1. PCM in embryos subjected to reduced RA reach control cranial positions by early neurula (st. 13). Embryos treated with citral or DEAB were incubated to early neurula stage (st. 13) and the expression pattern of *gsc* (A-C) or *chrd.1* (D-F) was determined by *in situ* hybridization. (A,D) Control. (B,E) citral. (C,F) DEAB. Dorsal view. The rostral limit of each gene matches the controls at this stage, however, the *chrd.1*-expressing notochord domain is shorter compared to controls.
